# Evaluating Zygomatic Implants in Orbital Floor Complications: A Clinical and Anatomical Study

**DOI:** 10.1007/s12663-024-02403-1

**Published:** 2025-01-09

**Authors:** Rafal Zielinski, Jakub Okulski, Wojciech Simka, Jerzy Sowinski, Jan Łoś, Marcin Kozakiewicz

**Affiliations:** 1 Stomatologia na Ksiezym Mlynie, Lodz, 16D Tymienieckiego, 90-365 Lodz, Poland; 2 Department of Maxillofacial Surgery, Medical University of Lodz, 113st Zeromskiego, 90-001 Lodz, Poland; 3 Faculty of Chemistry, Silesian University of Technology, 44-100 Gliwice, Poland; 4 Private Dental Clinic, Tetmajera 3A rd, 05-080 Izabelin C, Poland; 5 Miladent Private Dental Clinic, Gdańsk, Poland

**Keywords:** Zygomatic implants, Anatomy of orbit, Zygomatic orbital floor parameter

## Abstract

**Background:**

One of the most critical complications that can occur after placing zygomatic implants is the accidental penetration of the patient's eye socket by a drill or implant. This article aims to find some relationships between the anatomical features of the lower orbital wall, different configuration of zygomatic implants, and other factors when planning the placement of zygomatic implants.

**Methods:**

A total of 81 patients underwent zygomatic implant procedures, receiving different combinations of implants. These included four zygomatic implants along with one or two conventional implants (Group I), four zygomatic implants alone (Group II), two zygomatic implants paired with four conventional implants (Group III), or three zygomatic implants alongside one or two conventional implants (Group IV). The aim of the study was to describe the complications and clinical outcomes of treatment in 81 patients who received zygomatic implants.

**Results:**

The following parameters were statistically significant between all four groups of patients: height and distance of two zygomatic implants in the zygomatic bone; surgery type and duration; the distance between zygomatic implants in the zygomatic bone; and zygomatic orbital floor (ZOF) classification at the left side showed significant differences; intramaxillary insertion of zygomatic implants reduced the rupture of Schneiderian’s membrane; the average of zygomatic implants’ length in all groups was 42.8 mm.

**Conclusions:**

Not damaging the ZOF is profoundly important for preventing orbital damage during osteotomy for zygomatic implants and should be measured before every surgery.

## Introduction

Zygomatic implants are an effective treatment option for patients with severe maxillary atrophy, offering a graftless alternative to conventional implants, which often require bone augmentation. Unlike traditional dental implants, zygomatic implants anchor in the zygomatic bone, providing stability for prosthetic rehabilitation in cases where standard bone volume is insufficient. This technique is particularly beneficial for patients unwilling or unable to undergo complex bone grafting procedures. Insertion of standard dental implants is a more simple, faster, and less invasive method than zygomatic implants, but subperiosteal or zygomatic implants could be the method of choice in some individual cases. Such cases include patients with maxillary defects after tumor resection in whom microvascular reconstruction is not feasible [1–4].

Knowledge of the preoperative anatomy around the zygomatic bone (ZB) of a particular patient, such as the brain, eyes, nerves, and blood vessels, is crucial for performing safe surgery. One of the most critical complications associated with zygomatic implant placement is the risk of orbital penetration, particularly when the anatomy of the zygomatic bone and orbital floor is not adequately assessed [5–7]. The anatomical proximity of the orbit to the zygomatic bone makes accurate planning and placement of these implants crucial for preventing iatrogenic damage to the orbital contents, including the Schneiderian membrane and extraocular muscles.

Recent advancements in surgical planning, such as computer-aided design (CAD) and guided surgery techniques, have aimed to improve implant placement accuracy and minimize complications. However, these technologies are not universally accessible, and many clinicians still perform free-hand zygomatic implant placement. Understanding the anatomical variations in the zygomatic bone and orbital floor, and how they impact implant placement, is essential for achieving safe and successful outcomes in free-hand procedures.

The primary objective of this study is to compare different configurations of zygomatic implants in terms of their placement within the zygomatic bone, the orbital floor, and the maxillary sinus. By examining key variables such as implant length, position, and their relationship to anatomical landmarks, we aim to provide insights into how these factors influence surgical outcomes and complication rates. Specifically, this study introduces and applies a novel classification system—the Zygomatic Orbital Floor (ZOF) classification—to categorize the depth of the orbital floor and assess its influence on implant placement safety.

The study includes 81 patients treated with various configurations of zygomatic implants, with or without supplementary conventional implants. These configurations are compared to determine the impact of implant number and placement on surgical success, complication rates, and long-term outcomes. By focusing on the anatomical relationships and implant configurations, this study aims to provide a framework for safer zygomatic implant surgery, especially in cases of complex maxillary atrophy.

## Materials and Methods

### Patients

Eighty-one patients with moderate or severe maxilla atrophy received treatment between 2010 and 2017 in a privately owned Polish dental implant clinic, Stomatologia na Ksiezym Mlynie, Lodz. Check-up visits with follow-ups continued until 2023. All patients underwent cone-beam computed tomography (CBCT) before and after surgery. No template or planning software was used for any patient, with 100% of implants inserted free-hand.

All patients were treated by the same operator, who had no specialty in maxillofacial surgery but had vast expertise in dental implants and had undertaken many zygomatic implant courses worldwide. Temporary screw-retained acrylic bridges were delivered within 24 h in 100% of patients.

Exclusion criteria of patients were smoking and diabetes mellitus, whereas inclusion criteria was maxilla atrophy Cawood and Howell class IV, V, or VI.

**Fig. 1:**
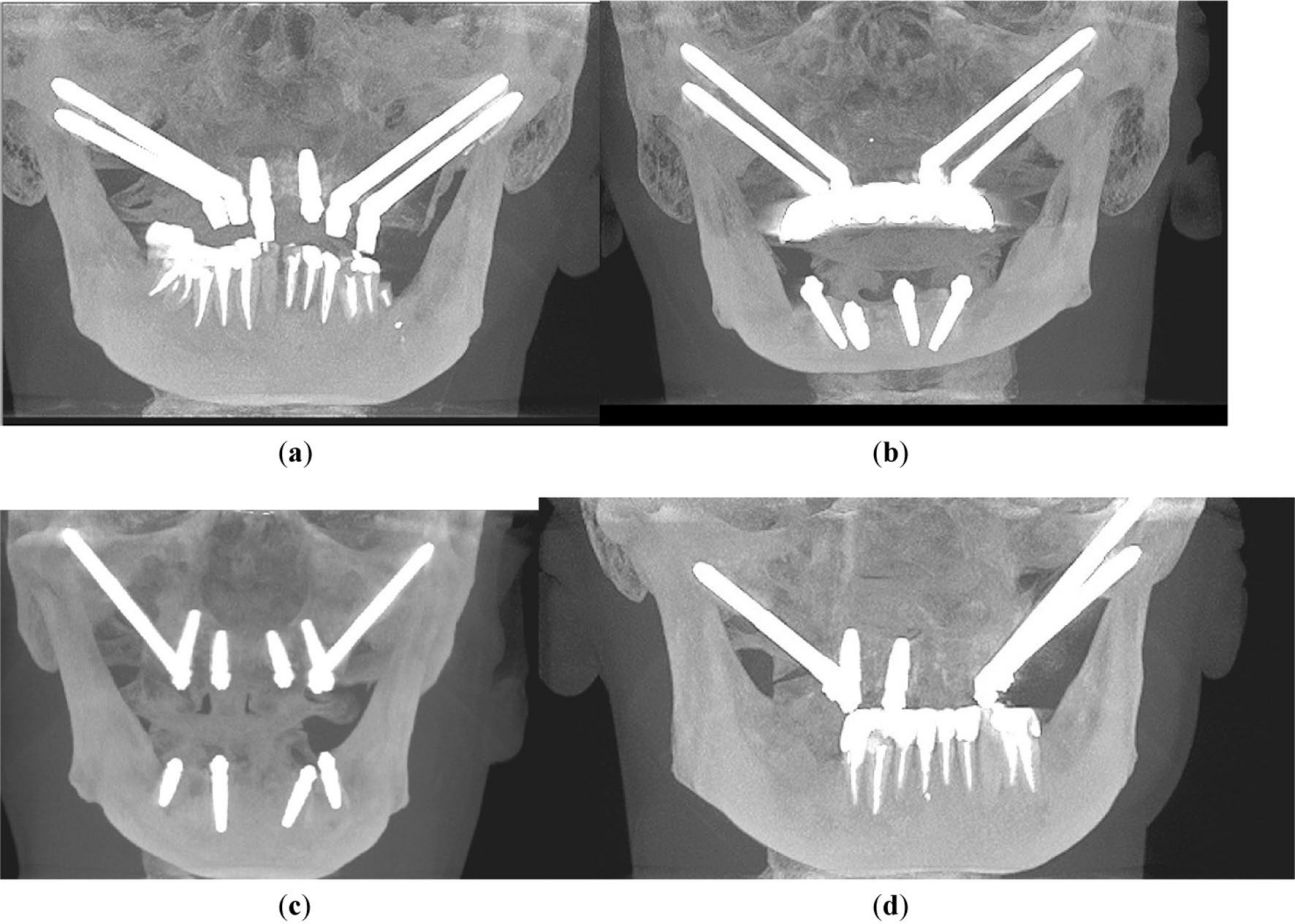
In the figure there are some representative CBCT photos for each group: **a** group 1—4 zygomatic implants + 1 or 2 conventional implants; **b** group 2—4 zygomatic implants; **c** group 3—2 zygomatic + 4 conventional implants; and **d** group 4—3 zygomatic + 1 or 2 conventional implants

Patients were divided into four groups with varying zygomatic implant configurations. Allocation to the group was performed on the basis of bone amount. When possible, additional bone root implants were inserted for the patient. When there was not enough bone on alveolar process, zygomatic implants were inserted. Group 1 (14 patients) received four zygomatic implants plus one or two conventional implants; In group 2 (15 patients) only four zygomatic implants have been inserted; group 3 (46 patients) had two zygomatic implants plus four conventional implants, and group 4 (6 patients) received three zygomatic implants plus one or two conventional implants (Table 1). In the Fig. 1 CBCT shows the groups among which authors divided patients.

**Table 1:** Patient groups on which implantation was performed

Type of surgery	Group 1	Group 2	Group 3	Group 4
Group description	Four zygomatic implants + one or two conventional implants	Four zygomatic implants	Two zygomatic implants + four conventional implants	Three zygomatic implants + one or two conventional implants
Number of patients	14	15	46	6

The protocol was reviewed and approved by an appropriate Institutional Review Board and that informed consent was obtained.

### Surgery

**Fig. 2:**
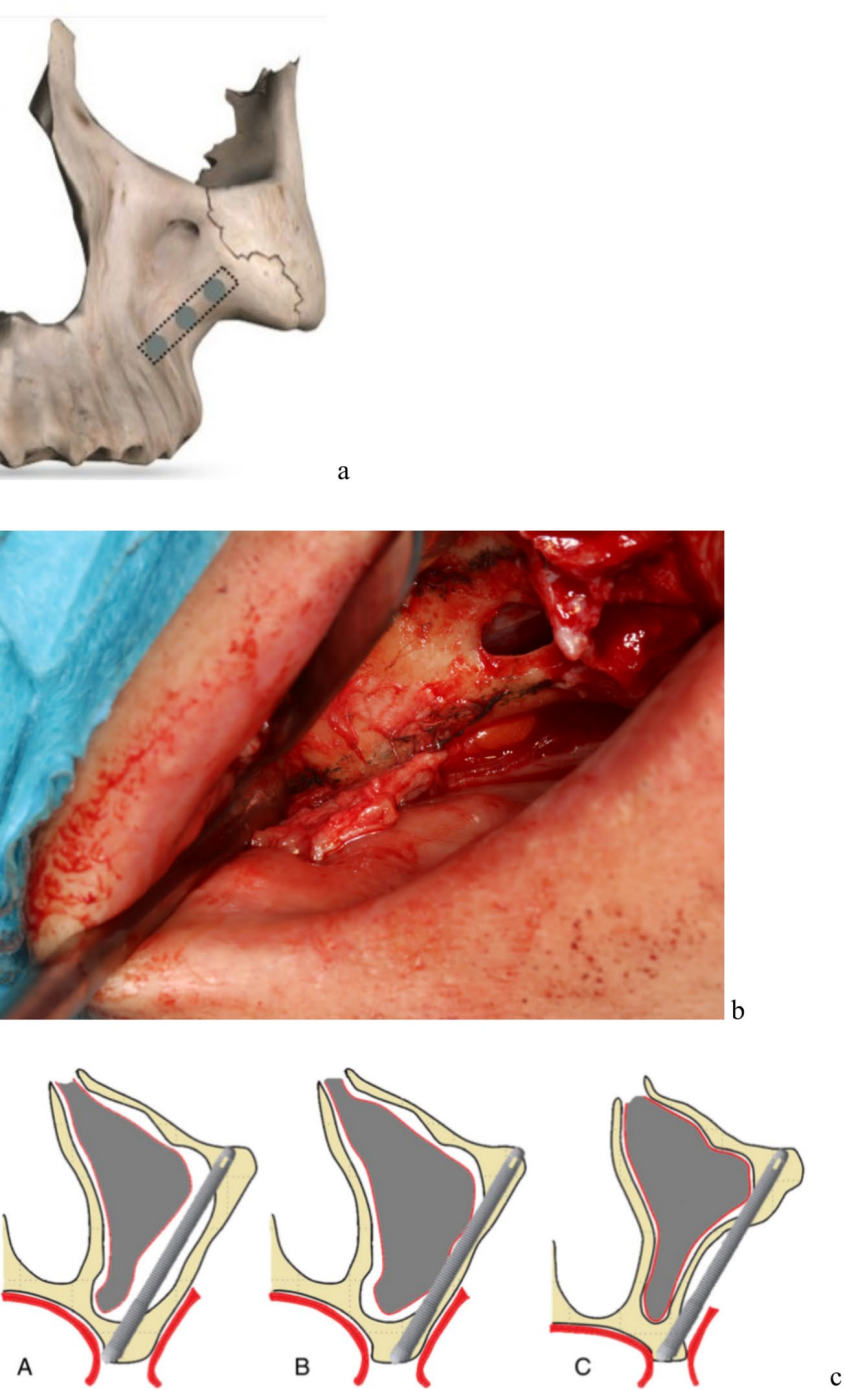
**a** picture presents a sketch of Stella’s method; **b** shows in real patient an antrostomy needed in Stella’s method; **c** shows the difference between: A Brånemark technique, B sinus slot technique—Stella’s method, C extrasinus technique

All patients were operated under general anesthesia with nasotracheal intubation, with additional local anesthesia—articaine with epinephrine—Citocartin 200 14 ml. The preferred method was the “sinus slot approach” described by Stella and Warner in 2000, which involves zygomatic implant placement that minimizes implant penetration into the maxillary sinus (Fig. 2) [8].

The operative technique commenced with a crestal incision extending from one maxillary tuberosity to the opposite tuberosity.

Some of the patients had residual teeth that demanded extraction just before osteotomy for placement dental implants and zygomatic implants. The alveolar crest and hard palate were exposed by raising a palatal flap. Continuing the dissection along the infra-zygomatic crest toward the ZB, the surgeon exposed the zygomatic region using a reverse Langenbeck hook while carefully locating the infraorbital nerve. Subsequently, a 4 × 3 cm window was meticulously created in the uppermost lateral aspect of the sinus wall, aligned with the extension of the infra-zygomatic crest, using a diamond round bur. After reflecting the sinus mucosa, direct visibility of the sinus roof was achieved, allowing for precise identification of the optimal point for drilling into the ZB. An implant handpiece was used to drill at 600 rpm to penetrate the crestal bone at the designated zygomatic implant entry area. During the surgery, the operator could see CBCT images focused on the zygomatic and orbital regions.

All zygomatic implants were Nobel Biocare 45° with a 4.3 mm diameter, and the length of all implants was registered. Surgery time was measured from the moment the patient fell asleep until the moment they awoke.

In all patients, the relationship between the location of the zygomatic implant and the maxilla and sinus was examined. The number of zygomatic implants was registered within regions 16, 13, 23, and 26, taking into consideration the intra and extrasinusal and intra and extramaxillary locations of the zygomatic implants. In all surgical procedures, the operator was standing on the right side of the patient’s head, and one dental assistant or another doctor helped.

The ORIS criteria were assessed separately for every zygomatic implant. Simple regression tests established the relationship between ZB height and width, while Mann–Whitney U tests compared the height and distance between two zygomatic implants in the ZB.

Final prosthesis was delivered after 6–8 months after the surgery.

### CBCT Standardization

An i-Cat CBCT scanner with a flat panel detector was used in all cases (Imaging Sciences International, LLC, PA, USA). The voxel size was 0.2 mm^3^ × 0.2 mm^3^ × 0.2 mm^3^, and exposure volume was set at 0.4 mm. Manufacturer-recommended settings of 80 kV and 5 mA were applied. The Frankfurt plane was used rather than the occlusal plane. The scan range encompassed the supraorbital edge to the mandible.

### Zygomatic Orbital Floor (ZOF)

Anatomy was thoroughly investigated by measuring the undercut of the lower and lateral walls of the orbit. Reference points in the frontal plane on the CBCT were the lowest point on the lower wall (B) and most lateral point (A) in the lateral wall of the orbit.

**Fig. 3:**
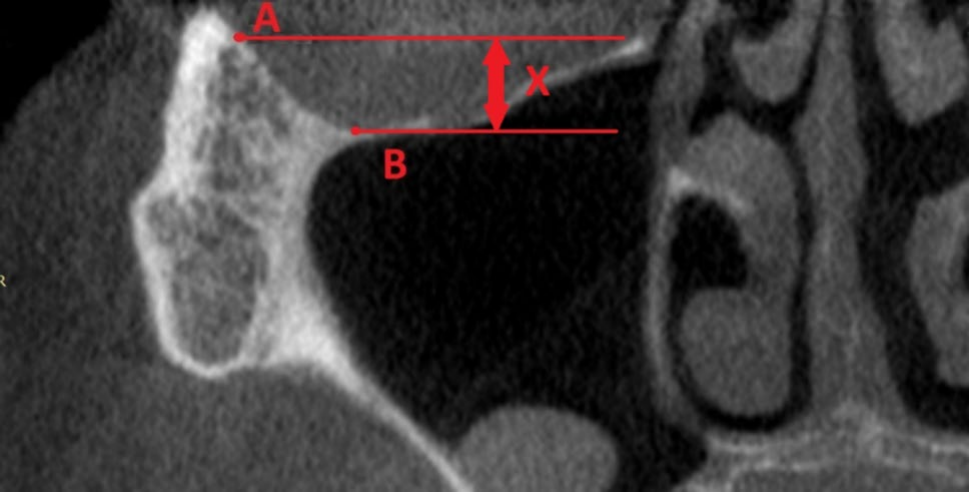
Reference points in the frontal plane on cone-beam computed tomography (CBCT) were the lowest (**B**) and the most lateral point (**A**) in the lower wall of the orbit

Two simple lines, parallel to each other and parallel to the horizontal plane, were prolonged, and the distance between them [x] was the authors’ own invention, the zygomatic orbital floor (ZOF) (Fig. 3).

The ZOF was divided into four classes:

Class I: 0–3 mm—flat.

Class II: 4–6 mm—moderate.

Class III: 7–9 mm—deep.

Class IV: more than 10 mm—extremely deep.

Simple regression and box and whisker plots were used to assess if ZOF classification was age-dependent. Analysis of variance (ANOVA) assessed if statistical significance existed between ZOF classification and anatomical structures—width on both sides and height of the ZB. The ZOF classification determines the angle of the zygomatic implants. The higher the ZOF classification, the lower the zygomatic implant must be situated in the zygomatic bone.

The ZOF classification was used before the surgery for surgical planning and the placement of zygomatic implants. By paying attention to the ZOF class, the operator avoided performing an osteotomy in the orbit. The measurements were utilized to plan not only the distance from the orbital rim to the osteotomy, which was marked with a pencil before drilling, but also to assist in planning the entire treatment, including the number of zygomatic implants on one side.

The ZOF classification has a significant impact on evaluating postoperative treatment because it not only makes the procedure safer but also helps determine the number of zygomatic implants, thus influencing the force distribution in the prosthodontic bridge.

## Results

Eighty-one patients (average age: 53 years, ranging from 54 to 70 years, with a standard deviation of 8.65) were examined and operated. The mean follow-up time was 8.8 years (min = 6 years and max = 13 years, SD = 2.5).

**Fig. 4:**
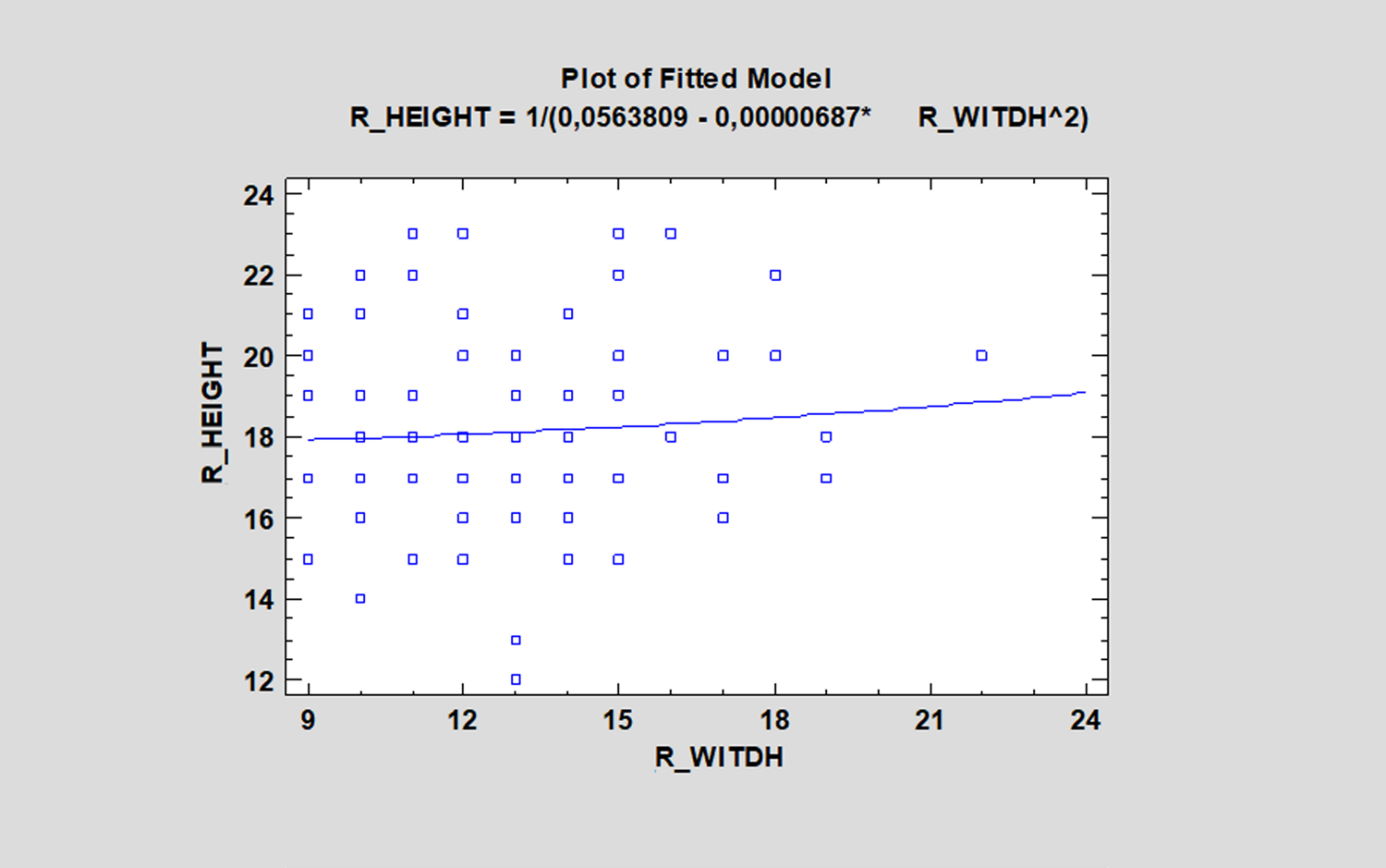
Simple regression showed no statistical significance between the height and width of the zygomatic bone (ZB) (*p* > 0.05)

Simple regression showed no statistical significance between the height and width of the ZB (*p* > 0.05). Such information is crucial for the operator, because it shows that a massive and wide ZB does not have to be high, and, thus, a surgeon should pay close attention to zygomatic orbital floor (ZOF) (Fig. 4). The height and distance between two zygomatic implants in the ZB was investigated, and the Mann–Whitney U test demonstrated the dependence (*p* < 0.05).

**Fig. 5:**
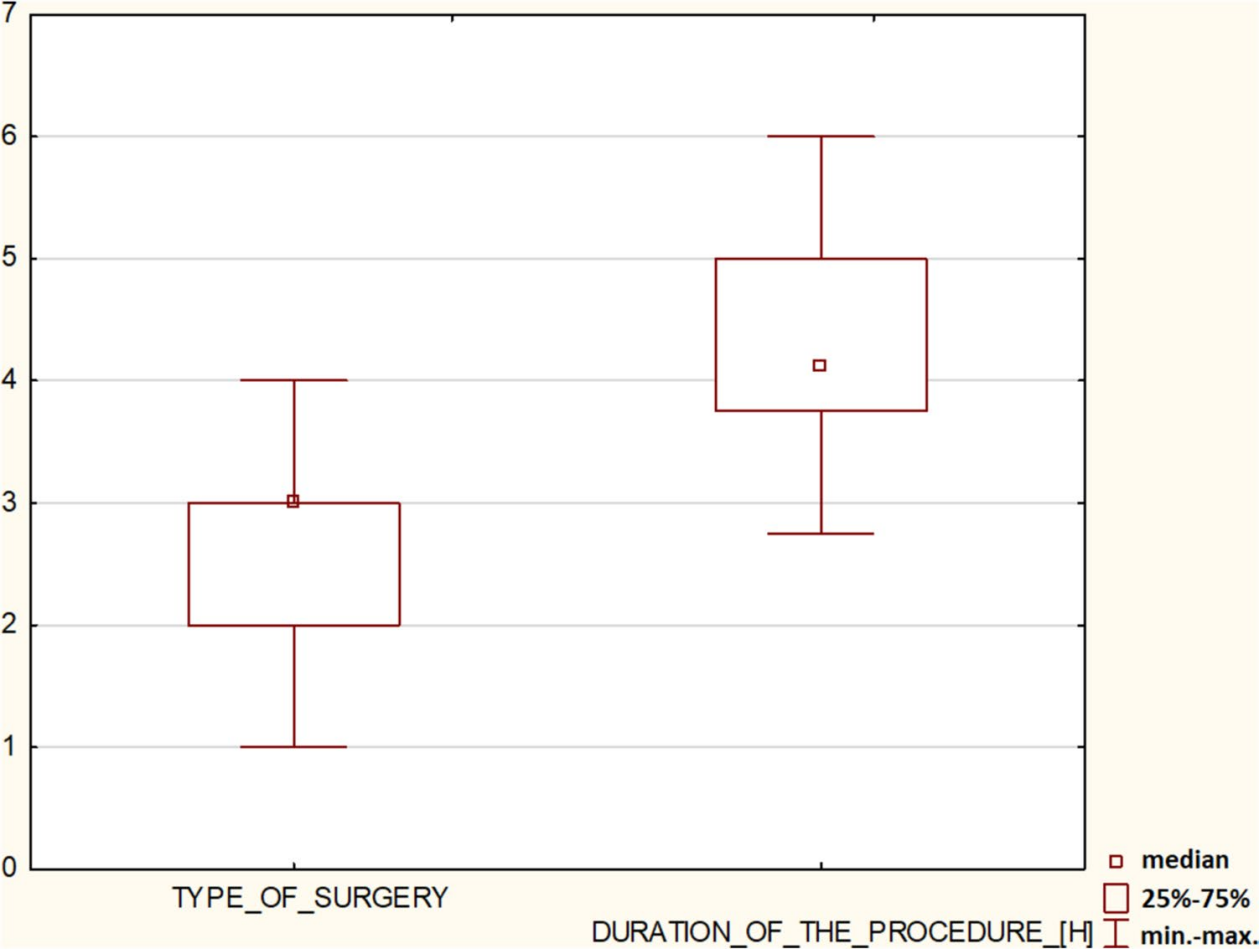
Procedure duration depended on the surgery type. Type of surgery: Group 1: (14 patients) four zygomatic implants plus one or two conventional implants; Group 2: (15 patients) four zygomatic implants; Group 3: (46 patients) two zygomatic implants plus four conventional implants, and Group 4: (6 patients) three zygomatic implants plus one or two conventional implants were screwed

The mean surgery time, including taking impressions and adjusting wax check-bite occlusion height during sleep, was 4.11 h (min = 2.75 and max = 6.75, SD = 0.85). Time was measured from the moment a patient went to sleep until the moment they woke up. The Mann–Whitney U test demonstrated dependence between surgery type and duration (*p* < 0.05) (Fig. 5).

The length of zygomatic implants for each group is presented in Table 2. The average of zygomatic implants’ length in all groups was 42.8 mm (min = 32.5 mm and max = 52.5 mm).

**Table 2:** Zygomatic implant length in each group

Type of surgery [groups]	Number of patients	Mean [mm]	SD [mm]	Min [mm]	Max [mm]
1	14	39.8	5	32.5	52.5
2	15	40.9	4.5	32.5	47.5
3	46	43.4	4.1	35	50
4	6	41.7	3.7	35	47.5
Total	81	42.8	4.7	32.5	52.5

In terms of the location of zygomatic implants in relation to the maxillary sinus [intrasinusally and extrasinusally], the following numbers of zygomatic implants were located in the following regions:

In region 16 [upper right molar]—67 and 14,

In region 13 [upper right canine]—31 and 2,

In region 26 [upper left molar]—61 and 20,

In region 23 [upper left canine]—19 and 3,

where the first value provided indicates the number of patients with implants placed intrasinusally, and the second value is the number of patients with zygomatic implants placed extrasinusally.

When it comes to the location of zygomatic implants in relation to the jaw [intramaxillary and extramaxillary] for the same regions, the following values indicate the numbers of patients in whom zygomatic implants were placed intramaxillary and extramaxillary, respectively:

For region 16 [upper right molar]—71 and 10,

For region 13 [upper right canine]—25 and 8,

For region 26 [upper left molar]—68 and 13,

For region 23 [upper left canine]—28 and 6,.

The difference in the positions of intrasinusal and extrasinusal zygomatic implants results from the anatomy of the patient's alveolozygomatic arch and the surgical method used. Stella's method was performed in all patients where anatomy permitted, and the alveolar process was preserved whenever it was possible (Fig. 2).

Table 3 shows the number of patients with intra/extramaxillar and -sinus zygomatic implants.

**Table 3:** Patients with intra and extramaxillary and sinus zygomatic implants

Number of zygomatic implants				
Extramaxilla	Intramaxilla	Extra sinus	Intra sinus	Region
10	71	14	67	16
8	25	2	31	13
13	68	20	61	26
6	28	3	19	23

ORIS (acronym of offset, rhinosinusitis, infection, and stability) scales for each region were: region 16: 69 Stage I, five Stage II, five Stage III, and two Stage IV. Region 13 had 26 Stage I, three Stage II, and four Stage III. Meanwhile, region 26 had 66 Stage I, seven Stage II, seven Stage III, and one Stage IV. Region 23 had 25 Stage I, three Stage II, four Stage III, and one Stage IV. [9]

The Mann–Whitney U test showed no statistical significance (*p* > 0.05) in terms of intra or extrasinusal or intra- or extramaxillary zygomatic implant placement and ORIS scale in all regions—13, 16, 23, 26.

**Fig. 6:**
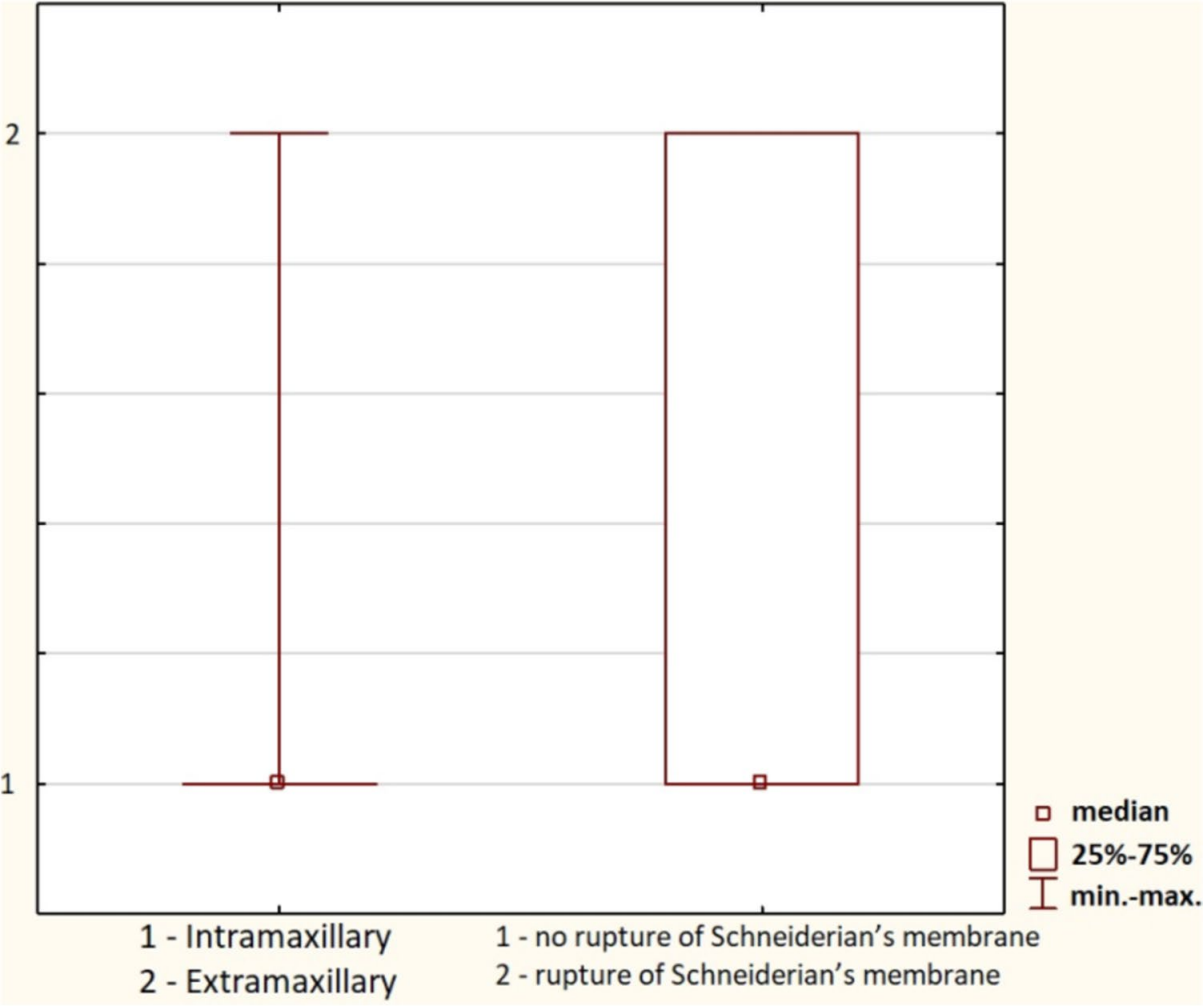
Intramaxillary insertion of zygomatic implants reduced the rupture of Schneiderian’s membrane (*p* < 0.05). 1—intramaxillary, 2—extramaxillary location of zygomatic implant; 1—no rupture of Schneiderian’s membrane, and 2—rupture of Schneiderian’s membrane

Schneiderian membrane was measured on CBCT. Intramaxillary insertion of zygomatic implants reduced the rupture of Schneiderian’s membrane (*p* < 0.05) (Fig. 6).

**Fig. 7:**
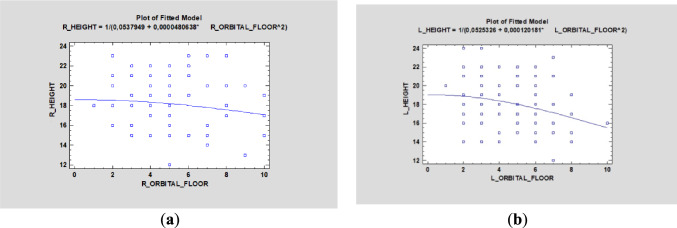
Simple regression analysis of zygomatic bone and orbital floor height [in mm] demonstrated statistical significance on the left side (**a**) (*p* < 0.05) but not on the right side (**b**) (*p* > 0.05)

**Fig. 8:**
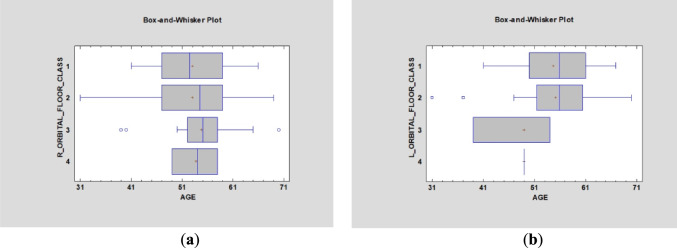
Box and Whisker plots were used to assess if zygomatic orbital floor classification depended on age (*p* > 0.05) on both sides (right—a and left—b)

The distance between zygomatic implants in the ZB and ZOF classification was statistically irrelevant on the right side, although the left side showed significant differences (*p* < 0.05) (Fig. 7). Furthermore, ZOF classification was not age-dependent on either side (*p* > 0.05) (Fig. 8).

**Fig. 9:**
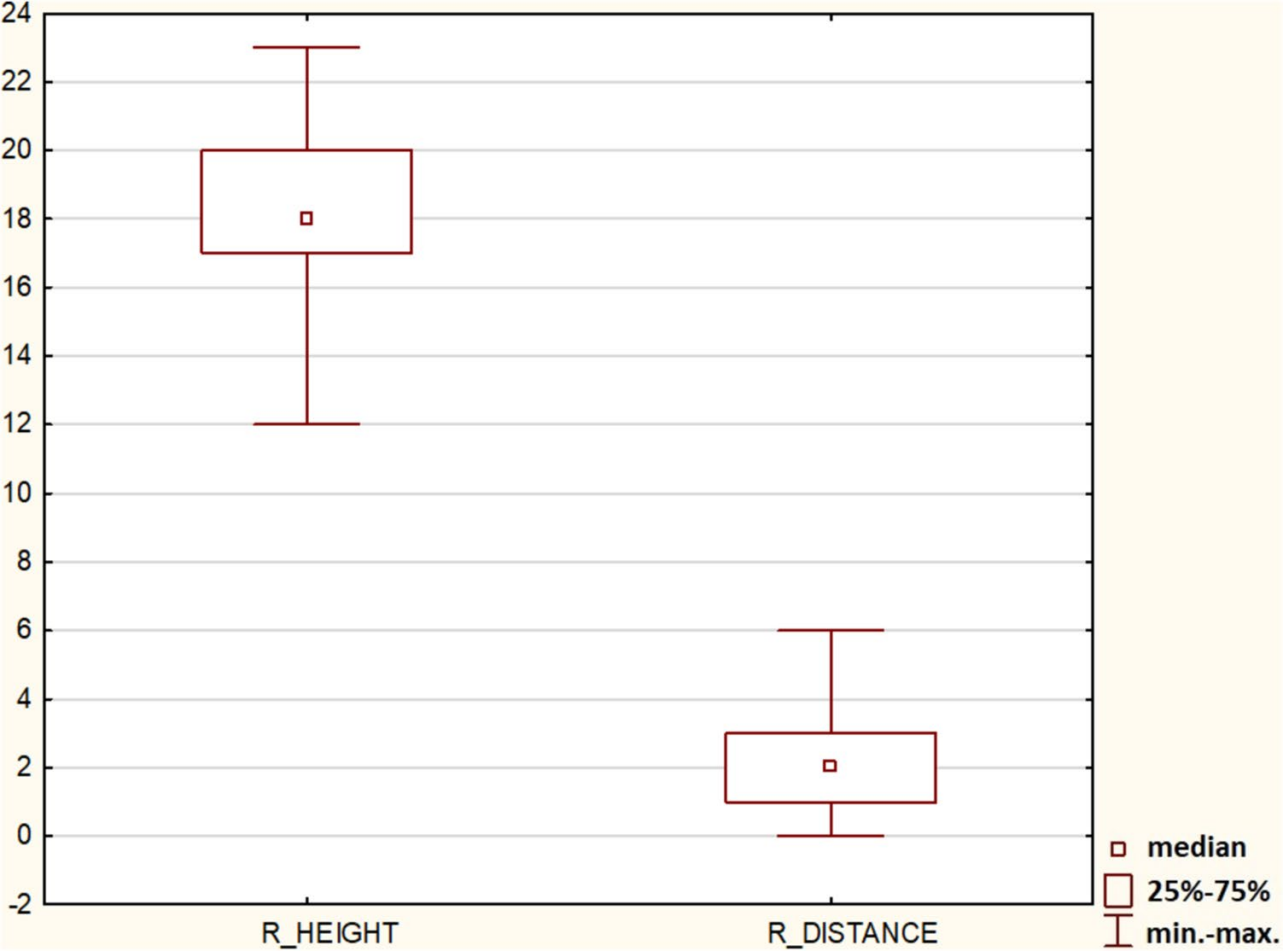
Height and distance between two zygomatic implants in zygomatic bone were investigated, and the Mann–Whitney U test demonstrated the dependence (*p* < 0.05)

The Mann–Whitney U test between the height and distance of two zygomatic implants in ZB proved statistically significant (*p* < 0.05) (Fig. 9).

## Discussion

The findings of this study provide critical insights into the relationships between zygomatic implant configurations and the anatomical structures surrounding the zygomatic bone and orbital floor. By introducing the zygomatic orbital floor (ZOF) classification system, we aimed to offer a novel tool for assessing the risk of orbital damage during free-hand zygomatic implant placement. The results of our study show statistically significant differences between implant configurations and their proximity to key anatomical landmarks, particularly in relation to the ZOF classification, which is consistent with the findings in prior studies. This section discusses how our results compare to existing knowledge and the clinical significance of these findings.

### Implant Configurations and Orbital Floor Proximity

Our results show that in patients with higher ZOF classifications (Class III and IV), implants were placed lower in the zygomatic bone to avoid the orbital floor, with a significant difference observed between the left and right sides (*p* < 0.05). Specifically, implants placed in patients with deeper ZOF classifications tended to be positioned at greater distances from the orbital rim, thus reducing the risk of orbital damage. This confirms that ZOF classification is an effective method for planning implant placement.

### Implant Length and Sinus Penetration

The study found that the average implant length across all groups was 42.8 mm (range: 32.5–52.5 mm). Implant length varied significantly between groups, with longer implants more frequently used in patients with more severe maxillary atrophy (*p* < 0.05). Interestingly, intramaxillary placement of zygomatic implants was associated with a reduced incidence of Schneiderian membrane rupture (*p* < 0.05), confirming that this technique is safer for avoiding sinus-related complications. Notably, implants placed intramaxillary reduced the risk of Schneiderian membrane rupture (*p* < 0.05), a finding consistent with the work of Grecchi E., et al. [10], who reported that intramaxillary placement results in fewer sinus complications compared to extrasinus placement. While Grecchi et al. used a guided surgical approach, our results show that free-hand surgery, when combined with proper anatomical assessments like ZOF classification, can minimize sinus-related complications as well. However, our study further expands on this by quantifying the specific relationship between implant length and anatomical safety, showing that longer implants, when placed correctly, do not increase the risk of sinus complications.

### ZOF Classification as a Novel Tool

The ZOF classification system introduced in this study provides a practical and effective method for assessing the depth of the orbital floor and its impact on zygomatic implant placement. Our results indicate that the deeper the ZOF classification, the greater the risk of complications if not properly accounted for. Specifically, patients with Class III or IV ZOF classifications benefited from lower implant placement in the zygomatic bone, reducing the likelihood of orbital penetration (*p* < 0.05). This supports the utility of the ZOF classification in clinical practice, particularly in cases where advanced guided surgery tools are not available. Previous research has largely focused on the use of CAD and guided systems to mitigate the risks of orbital damage [11]. While those studies advocate for guided surgery, which can reduce complications, the ZOF classification offers a practical alternative for surgeons who may not have access to advanced digital tools. Our findings demonstrate that by categorizing patients based on their orbital floor anatomy, clinicians can plan the angle and depth of osteotomies to minimize the risk of orbital complications.

Orbital penetration is a well-documented complication of zygomatic implant surgery, with studies by Mertens et al. [1] and Wu et al. [12] reporting cases of orbital damage in free-hand surgeries. Our study had no cases of orbital perforation, which can be attributed to the operator’s consistent use of preoperative CBCT planning and the avoidance of high-risk ZOF classifications. This contrasts with Krauthammer et al. [13], who reported extraocular muscle damage in a case of orbital penetration during free-hand surgery. Our findings underscore the importance of understanding the patient’s specific orbital anatomy through detailed imaging, even in the absence of digital guides, thereby reinforcing the clinical utility of ZOF classification as a protective measure. Furthermore, we have found that surgery duration correlated with both implant configuration and patient-specific anatomical challenges (*p* < 0.05). This reinforces the importance of individualized surgical planning based on precise anatomical measurements.

### Success Criteria of Zygomatic Implants—ORIS

Those who work with zygomatic implants concur that the success criteria for traditional implants do not directly apply to zygomatic implants. Thus, Aparicio et al. outlined four distinct criteria to be considered: offset of the definitive prostheses, rhinosinus condition, soft tissue infection, and stability. [9] Evaluating these criteria allows a practitioner to categorize a patient into one of the following five conditions:

• Success condition I: The optimal stage.

• Success condition II: A deviation from the norm without clinical impact.

• Success condition III: A borderline situation with clinically evident alterations, yet still treatable.

• Condition IV: Reflects a surviving implant supporting the prosthesis but not evaluated by the proposed criteria.

• Condition V: Represents implant failure.

#### Offset of the Prostheses [O]

**Fig. 10:**
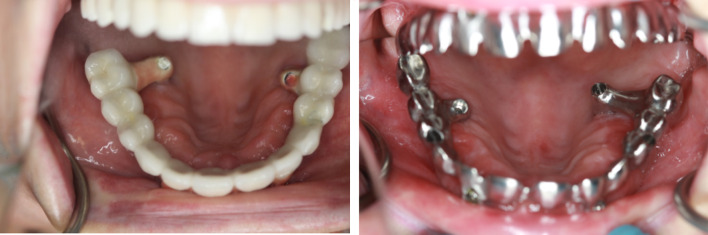
The emergence of zygomatic implants in the palate may lead to prostheses with "piping"

**Fig. 11:**
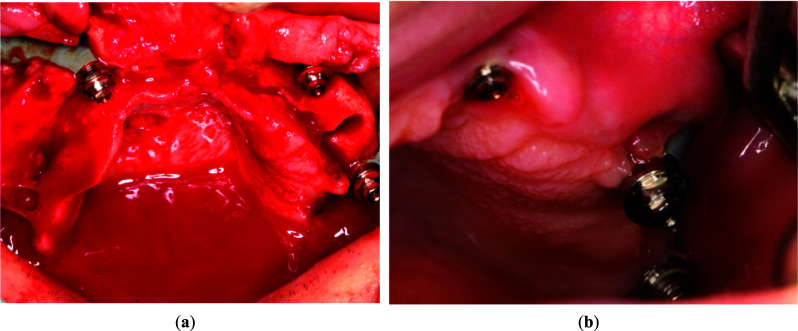
Implant screwed in region 26 screwed too buccally (**a**) resulted in implant removal after 2 years of surgical procedure (**b**). Lack of keritanized mucosa caused implant dehiscence

The emergence of zygomatic implants in the palate may lead to prostheses with "piping". Occasionally, a substantial dental bridge on the palatal side can cause discomfort, impacting speech, and limiting hygiene access (Fig. 10). Conversely, placing the implant too buccally can result in zygomatic implant failure (Fig. 11).

#### Rhinosinus Status [R]

The health and status of the sinuses are crucial and should be assessed both clinically and radiographically, as detailed in the Lanza and Kennedy survey (Table 4) (L-K survey) and the Lund-Mackay system. We suggest using the Lund-Mackay.

**Table 4:** Lanza and Kennedy table shows rhinosinusitis criteria necessary for assessment for ORIS

Lanza and Kennedy Task Force on Rhinoosinusitis Criteria for the Diagnosis of Rhinosinusitis		
Major criteria	Minor criteria	Diagnosis of rhinosinusitis requirements
Facial pain or pressure	Headache	Two or more major criteria
Facial congestion or fullness	Fever (non acute)	One major and two or more
Nasal obstruction	Halitosis	Purulence on nasal examination
Purulent discharge	Fatigue	
Hyposmia or anosmia	Dental pain	
Purulence on examination	Cough	
Fever (acute only)	Otalgia or aural fullness	

staging system, a validated scoring system recommended by the task force on rhinosinusitis for research outcome. The radiological test includes six regions: anterior ethmoid; posterior ethmoid; maxillary; frontal; sphenoid; and osteomeatal complex. Each region is given a score of 0, 1 or 2—0: no abnormality; 1: partial opacification; and 2: complete opacification. The ostiomeatal complex (OMC) is scored as either 0 (not obstructed) or 2 (obstructed). Any scan with a score of > 0 would be considered an abnormal or ‘positive’ scan. [14, 15]

#### Periimplant Soft Tissue Condition [I]

Implant exposure resulting from soft tissue dehiscence can cause partial visibility of the implant. This also reveals the delicate bone layer situated between the neck of the implant and the sinus cavity. Over time, this exposed bone tends to undergo more resorption, generally without inducing pain. If this condition remains unchecked, especially in critical areas with minimal bone thickness, it could eventually form a pathway to the sinus. The accumulation of bacterial films, particularly pronounced on implants with threads or rough surfaces, can lead to sustained inflammation of the soft tissue surrounding the implant. Such inflammation can further complicate matters by hastening bone remodeling, which may then result in a range of issues. These include potential communication with the sinus, esthetic problems, mucositis, and in severe cases, cellulitis.

#### Stability [S]

Confirming osseointegration via the clinical mobility test is a widely accepted method, and this has been extended to zygomatic implants as well. Nevertheless, when examining a zygomatic implant, variations in implant stability can be evident. For instance, when non-axial forces are applied to an externally positioned zygomatic implant anchored in suboptimal zygomatic bone quality, slight movement might be detected. It is essential to understand that this movement doesn't always align with clinical symptoms or pathological signs. Such movement is attributed to the bone’s elasticity counteracting the lateral force applied externally on the protruding head of the implant, inducing bending stress. This mobility should not be of concern if other symptoms are absent, and it usually diminishes after fastening the superstructure using screws. In essence, minor, pain-free mobility can be attributed to the elastic nature of the anchoring zygomatic bone when the implant faces lateral force externally. A Grade I success indicates an absence of detectable movement, Grade II suggests minimal discernible movement, while Grade III denotes noticeable movement without evidence of loss of osseointegration. On the other hand, failure is characterized by apparent movement combined with rotation or pain. Any observed rotational movement of the implant should be unequivocally considered a sign of implant failure, regardless of accompanying discomfort. The ORIS scale emerges as the most pertinent tool for evaluating the success of zygomatic implants.

### A Surgical Template

Only highly experienced and skilled surgeons should undertake the placement of zygomatic implants to minimize the occurrence of postoperative complications, particularly those affecting the adjacent anatomical structures, such as the orbit [16, 17]. Traditional implants differ from zygomatic implants in many aspects [18–22].

Even though all of the implants in the study were inserted free-hand, with only the help of a three-dimensional (3D)-printed skull, inferior alveolar nerve and orbit damage did not occur. However, some articles describe the advantages of fully guided surgical procedures for zygomatic implants. Thanks to surgical templates, the risk of dysesthesia or orbit penetration should be low, even in less experienced surgeons. By superimposing preoperative digitally planned zygomatic implants and postoperative CBCT.stl files, the accuracy of using the template was examined. Many authors state that surgical templates minimize the risk during implant placement, and make implant positioning more precise [23–25].

Flügge et al. claim that preoperative radiological assessment can be imprecise and endanger the patient’s health. However, based on our numerous patient groups, we must disagree with the statement when the osteotomy channel is visible during surgery, with the implant probe palpable on the skin in the zygomatic region.

The main drawbacks of surgical templates for zygomatic implants are the price, the necessity for specially designed software, delivery time, and the time required to design and melt them. All articles on surgical templates for zygomatic implants contain information on the price of the device. Even though the results of discrepancies between the planned and final position of zygomatic implants when using templates are very promising, further investigations should be performed. Indeed, operators must remember that a template is just a tool that might also be faulty. Gao et al. compared the final zygomatic implant position between free-hand and guided-off surgery with the planned position and found big differences, especially in angular positions (*p* < 0.05). Another study showed the difference between plan and post-surgery angulation ranging from 0.5 to 14°, with an average of 5°. [26] Regarding zygomatic implants, some authors describe up to 2.5 mm linear discrepancies and 3 degrees in angulation [27]. In the current study, the distance between the zygomatic implant and the orbit was about 1 mm in some cases, and given that a 2-mm deviation could have occurred, orbit perforation was possible. This conclusion shows that surgical experience and knowledge of patient’s anatomy, especially the orbital wall localization and ZB height and width, are of paramount importance.

Precise pre-surgical planning, with or without a template, and a strict drilling protocol are essential for the zygomatic implant procedure. Previous studies highlighted the risk of orbital damage, and some reported specific incidents. Duarte et al. conducted the implantation of quad-zygomatic implants in 12 patients, resulting in two cases of orbital cavity penetration [28]. Similarly, Davo et al. reported one instance of orbital cavity penetration out of 17 cases, but no permanent ocular damage was observed in any of these incidents [29]. Krauthammer et al. reported permanent extraocular muscle damage [7].

To address this risk, Wu et al. [12] proposed a new technique involving the pre-exposure of the inferiolateral orbital rim by an ophthalmologist. However, this method demands an experienced ophthalmologist on the surgical team and makes the procedure more aggressive. [30] We decided not to use surgical templates in order not to dull the vigilance of the operating doctor and to avoid subjecting the patient to additional costs.

### Stella’s Method

**Fig. 12:**
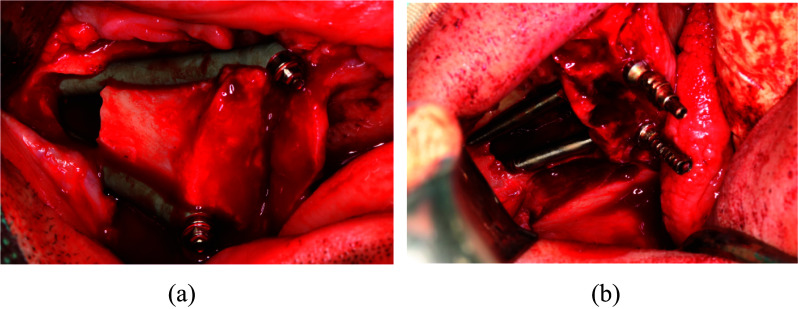
**a** Intrasinusal and extramaxillary and **b** intrasinusal and intramaxillary implant placement according to the Stella technique

Another risk of zygomatic implants is maxillary sinus inflammation. To prevent or at least reduce this risk, zygomatic implants should be placed extrasinusally or extramaxillary. Such an approach to osteotomy preserves the Schneiderian membrane. In the extramaxillary approach, multi-units attached to the implant nest are positioned at or near the top of the alveolar crest in the most convenient location. [31, 32] The main disadvantage of this method is the high risk of dehiscence of the zygomatic implant within the maxillary sinus as a result of Schneiderian membrane’s rupture during surgical procedure. However, its primary advantage is the preservation of alveolar bone. As a result, the risk of dehiscence is much lower because the gingiva lies directly on the bone and not on the implant. With this method, however, there is a risk that the position of the zygomatic implant head may not be ideal. The authors determined that the risk of palatal placement of the zygomatic implant head poses less danger in the long run than the dehiscence of the zygomatic implant. Thus, to prevent or at least mitigate this risk, the authors of the article opted to place implants using Stella's method (Fig. 12).

### Zygomatic Orbital Floor (ZOF)

Instead of this invasive method, Zielinski et al. suggested improved orbital anatomy investigation, especially for the aspects of ZOF classification, as described in their article. We demonstrated that extramaxillary insertion of zygomatic implants was an appropriate method for prosthodontic reasons, as the mounting screws on the bridge are located on the occlusal surface of the tooth; however, the more external from maxilla the higher risk of dehiscence of an implant. Moreover, the intra/extrasinusal or intra/extramaxillary placing of zygomatic implants in majority cases did not influence the ORIS scale. The distance between two zygomatic implants in the zygomatic bone (ZB) was statistically different on the left side (*p* < 0.05) but not on the right side (*p* > 0.05). The lowest risk of orbit’s damage in ZOF classification is class I: 0–3 mm—flat. In class I, the orbit’s undercut is minimal so during osteotomy between zygomatic bone and alveolar crest, there is straight line without orbit in the drill’s path.

A possible reason was the position of the operator, as they were always on the right side of the patient’s head and did not use a template.

### Oncology

Zygomatic implants are also helpful in patients following a hemi-maxillectomy. ^27^ Smaller defects in the oral cavity can be managed using dental obturators. However, larger defects, especially those that need bone support, are best treated with microvascular grafts. The donor soft tissue flaps are the fasciocutaneous radial forearm flap or the anterolateral thigh flap. For more extensive defects, alternatives such as the fibula graft [33, 34], the iliac crest flap [35], and the scapular flap [36] have been proposed.

In patients with unstable health status, the method of choice is a dental obturator. However, this approach leaves much to be desired, as much depends on retention, sealing, and the area of remaining teeth. Xerostomia induced by radiation or edentulism also influences poor retention. Using zygomatic implants after hemi-maxillectomy and conventional implants if there is adequate bone on the healthy side enhances obturator stability.

### Strengths and Limitations and Clinical Significance

#### Strengths

*-* The use of a large cohort of 81 patients over a long follow-up period (up to 13 years), which enhances the study's reliability. Long-term follow-ups give more reliable insights into the durability and success of implants, helping clinicians make informed decisions.
–The novel focus on zygomatic orbital floor (ZOF) classification in relation to implant placement safety, which adds to the understanding of preventing orbital complications. This adds a new dimension to the planning and safety of zygomatic implant placement, potentially reducing complications related to orbital damage.
**-** The study includes four distinct patient groups with different zygomatic implant configurations, providing a broad comparison of implant techniques and their clinical outcomes. This allows for a better understanding of which configurations might be more effective under certain anatomical conditions.
- All procedures were performed free-hand by the same experienced operator, ensuring consistency in the surgical approach. This consistency eliminates variability that can occur with multiple operators, making the results more reliable for this specific technique.
- The use of CBCT scans before and after surgery provides precise anatomical data for implant placement and outcome assessment. CBCT imaging allows for accurate measurements and detailed analysis, improving the study’s reliability in evaluating complications and outcomes.

#### Limitations

-The study was conducted by a single operator, which could introduce bias related to the surgeon’s technique and experience. The results may not be generalizable to other surgeons, especially those with less experience in zygomatic implant placement.
-The study lacks a randomized control group, and patients were allocated to different groups based on anatomical conditions rather than randomization. This can introduce selection bias, as certain anatomical features may influence outcomes differently across groups.
-The study did not incorporate digital planning tools or advanced guided surgery techniques, which are becoming more common in zygomatic implantology. This could make the findings less applicable to modern practices that increasingly rely on digital planning for precision.
- Surgical templates or guided surgery were not used, which could have led to variations in implant placement accuracy. This could limit the reproducibility of the technique, especially for less experienced surgeons, and introduces a potential risk of complications in inexperienced hands.
- The study was conducted in a single private clinic with a specific patient demographic. The findings may not be generalizable to other populations, particularly in different geographic regions or clinical settings.

#### Clinical Significance

The study provides critical insights into the anatomical considerations and technical nuances of zygomatic implant placement, particularly in relation to orbital floor anatomy. The ZOF classification could serve as a valuable tool in clinical practice to improve the safety of these complex procedures.

## Conclusions

Zygomatic implants should be performed under general anesthesia in a safe environment but the operator should be highly aware of the anatomy of the particular patient’s orbit to avoid its perforation; thus, ZOF parameter should be taken into consideration. Although virtual planning software and guided surgery splints might not be essential for the placement of zygomatic implants, many authors advocate for their use, as they can minimize risks and enhance precision. Regardless, intraoperative examinations, such as visual and palpable assessments of the maxilla, orbital rim, and ZB, are paramount for ensuring a safe surgical procedure. In cases of urgent orbital complications, such as iatrogenic retrobulbar hemorrhage, the operator should be able to perform immediate lateral canthoplasty and cooperate with the maxillofacial, oculoplastic, or ophthalmologist departments.

Both extra and intramaxillary zygomatic implant anchorage are surgically and prosthodontically acceptable, and the placement of zygomatic implants depends on an individual’s anatomy.

In Stella’s method, patients should take X-ray of maxillary sinus in order to examine health status of sinus due to intrasinus path of the zygomatic implants.
